# Vr exposure in cbt is effective and efficacious treatment for simple phobia (flight phobia)

**DOI:** 10.1192/j.eurpsy.2021.317

**Published:** 2021-08-13

**Authors:** A. D’Ambrosio, C. Tonelli, V. Martini, C. Ambrosio

**Affiliations:** Vre-cbt Psychotherapy, CBT CLINIC CENTER srl, Napoli, Italy

**Keywords:** phobia, flight phobia, virtual reality, VRE-CBT

## Abstract

**Introduction:**

The virtual environment with realistically rendered fear-inducing stimuli is enough to conduct VR exposure therapy (VRE), although the total control over the virtual environment also enables presentation of stimuli, contexts, and tasks not possible in in vivo exposure therapy (i.e. flight etc.)30 randomized controlled trials revealing high efficacy and effect sizes comparable of VRE-CBT to in vivo exposure therapy. Aerophobia is a very frequent limitation and affect 25% of the population and 30% of the subjects who fly make habitual use of anxiolytics.

**Objectives:**

The aims of this study is to show that conducting VR exposure in CBT for simple phobia (flight phobia) is effective and is an efficacious treatment for fear and anxiety,Vs other treatments.

**Methods:**

Participants (n = 39; age between 19 and 60 years) in the active arms received individual CBT VR exposure for six sessions and outcome was assessed with questionnaires: MSPS;Rathus Assertiveness Scale (RAS); HAM-A; QMAV; QSAV – (Flying fear); QoL INDEX and a behaviour avoidance test (really take the plane). Wilcoxon tests was using for the statistical analysis.

**Results:**

36 subjects managed to take the plane at the end of treatment and the results obtained showed a significant difference between “before treatment (T0) and after (T1)” with the exception of the Rathus test. All the SF-36 scales show a significant difference between “before-after”. 3 subjects was dropped out

**Conclusions:**

Using VR can be advantageous over standard CBT as a potential solution for treatment avoidance and as an efficient, cost-effective and practical medium of exposure.

**Disclosure:**

No significant relationships.

